# Effect of lactating sows’ diet supplemented with cactus (*Opuntia ficus-indica*) on feed intake and reproductive and productive post-weaning performances

**DOI:** 10.1007/s11250-018-1611-x

**Published:** 2018-05-09

**Authors:** Gerardo Ordaz-Ochoa, Aureliano Juarez-Caratachea, Rosa Elena Pérez-Sánchez, Héctor Eduardo Martínez-Flores, Juvenal Esquivel-Cordova, Ruy Ortiz-Rodríguez

**Affiliations:** 1 Instituto de Investigaciones Agropecuarias y Forestales, Universidad Michoacana de San Nicolás de Hidalgo, Km 9.5 Carretera Morelia-Zinapécuaro, Tarímbaro, Michoacán Mexico; 2 Facultad de Agrobiología “Presidente Juárez”, Universidad Michoacana de San Nicolás de Hidalgo, Paseo Gral. Lázaro Cárdenas y Berlín S/N Col Viveros, 60170 Uruapan, Michoacán Mexico; 3 Facultad de Químico Farmacobiologia, Universidad Michoacana de San Nicolás de Hidalgo, Tzintzuntzan 173, Matamoros, 58240 Morelia, Michoacán Mexico; 4 Facultad de Medicina Veterinaria y Zootecnia, Universidad Michoacana de San Nicolás de Hidalgo, Av. Acueducto S/N esquina Tzintzuntzan, Col Matamoros, 58130 Morelia, Michoacán Mexico

**Keywords:** Sow, Hypophagia, Lactation, Feed, Weight loss

## Abstract

The effect of cactus (*Opuntia ficus-indica*) added to the diet of lactating (21 days of lactation) sows on voluntary feed intake, and its impact on the productive and reproductive post-weaning performance was evaluated. Data collected of 72 farrowings from 37 hybrid sows were analyzed during 12-month period. The sows were divided into two groups: (i) control group (CG; *n* = 18 sows), sows fed only with commercial feed, and (ii) experimental group (EG; *n* = 19 sows), sows fed with commercial feed plus cactus supplement. The variables evaluated were blood glucose (BG), daily feed intake (DFI) and total feed intake (TFI), loss of body weight (LBW), weaning-estrus interval (WEI), and subsequent litter size (SLS). Data analysis was carried out using fixed effects models. A nested effect was found for farrowing number (FN) into of group and an interaction group × season on the analyzed variables (*P <* 0.001). EG observed lower levels of BG with 47.0 ± 7.9 mg dL^−1^ pre-prandial and 56.1 ± 5.9 mg dL^−1^ post-prandial at the 10th day of lactation (*P <* 0.05). DFI and TFI were higher in the sows of the EG independently of the FN and season (*P <* 0.05). No differeces were observed on the nested effect of FN into group on the levels of BG (*P <* 0.05). Autumn showed the higher TFI: 121.4 kg^−1^ sow^−1^ (*P <* 0.05). Sows from CG 3rd farrowing and from EG 4th farrowing observed higher LBW (13.8 and 6.9%, respectively) (*P <* 0.05). Summer showed a higher LBW with 12.7% for CG and 8.2% for EG (*P <* 0.05). EG showed a lower WEI (5.5 days) and greater SLS up to 1.8 piglets more depending upon the season (*P <* 0.05). The lactating sow’s diet supplemented with cactus can counterbalance the negative effects of lactational hypophagia due to reduction on levels of BG during lactation and an increase on DFI and, therefore, improves performance of LBW, WEI, and SLS.

## Introduction

The importance of the feeding of the sows, during lactation phase, resides in its effect on the productivity of the herd (Xie et al. 2015). Several authors have pointed out the effect of quantity and quality lactating sow feed intake on increased milk production (Hansen et al. 2012) and weight of litter at weaning (Tan et al. 2015). Others also have mentioned other effects such as less mobilization of body reserves (Cools et al. 2014) and a decrease in the weaning-estrus interval (Gunn et al. 2014). In addition, Rempel et al. (2015) pointed out a higher productive performance of sows in the next cycle. However, Suriyasomboon et al. (2006) indicate that the season of year is a factor that is also associated with the productivity post-weaning of the sow, since the increase in environmental temperature has a negative effect on reproductive indicators of the sow, either by acting directly on ovarian function or at the level of the hypothalamus-pituitary-ovarian axis (Segura et al. 2015). Likewise, the elevated temperatures have a negative effect on the volume of ejaculate, total sperm count, and morphology of the sperm of the boars, which will be reflected in smaller size of subsequent litter (Suriyasomboon et al. 2004).

Thereby, environmental factors and age (farrowing number) of sows can limit the optimal feed intake for the lactating sow and the physiological aspects of the sow during the lactation phase (Olsson et al. 2011; Yoder et al. 2012). However, strategies for the control of environmental factors have not proven to have a total efficacy to achieve the optimal feed intake of sows in lactation, because this variable (feed intake) during the lactation period is regulated mainly by the endocrinological and metabolic changes by those who pass the sows immediately after farrowing and during lactation, which are more accentuated in sows of 1st and 2nd farrowing compared with sows of three or more farrowings (Mosnier et al. 2010), because these (1st and 2nd farrowing sows) have higher nutrient requirements for growth, as they have not yet reached the size and weight of adults and have limited body reserves of proteins and fats. These increased demands for growth in addition to nutritional demands for milk synthesis may affect their capacity to return to estrus (Chansomboon et al. 2009; Segura et al. 2015). In addition, the primiparous sows have lower feed intake during lactation, which leads to greater weight loss, delay in posfarrowing ovarian reactivation, and therefore, increased weaning-estrus interval (Mosnier et al. 2010; Pérez et al. 2015a).

Among the metabolic alterations that affect the feed intake of the lactating sows emphasizes the increase of energetic substrates (glucose mainly) due to the insulin resistance that is presented in this stage; this is in response to the growth of fetuses and the preparation of the udder before the imminent lactation phase (Hansen et al. 2012). This phenomenon causes, in part, the detriment of the feed intake mainly during the first week of lactation, phenomenon known as lactational physiological hypophagy (Mosnier et al. 2010). The other component that generates the lactational hypophagy is endocrinological, and this is established during the hypergonodrotropic phase, specifically during the first 3 days post-farrowing (Yoder et al. 2012). As can be observed, the lactational hypophagy is an inherent physiological event of the sow in gestation (last third of gestation) and during the first week post-farrowing (Mosnier et al. 2010). From here, it is difficult to control and manipulate the feed intake of sows during the lactation phase.

It has been established (Jha and Berrocoso 2015) that the intake of dietary fiber in lactating sows improves the feed intake during the lactation phase, due to its favorable effects on the transit time of intake and the capacity of retention of water, aspects that take advantage of the microbiota of the digestive system of the sows, expressing itself, all this, in the improvement of the metabolic profile of the sows (Berrocoso et al. 2015). In humans, the dietary fiber of various foods, including cactus (*Opuntia* spp.), has been associated with an improvement in the metabolism of glucose, due to a positive effect on the synthesis of insulin (Deldicque et al. 2013). Thus, the ingestion of cactus by lactating sows regulates blood glucose levels during the first week post-farrowing and decreases insulin resistance (Ordaz et al. 2017) and, consequently, a diet supplemented with cactus reduces the effects of lactational hypophagia in lactating sows; since, cactus, apart from facilitating the synthesis of insulin due to its input of Ca^2+^ (Deldicque et al. 2013), contains dietary fiber (not digested or absorbed by gastrointestinal enzymes) that modifies the absorption of bile salts, colesterol, and glucose (Hsu et al. 2004). This is due to the pectin and mucilage found in the cactus, which produce food bolus viscosity and reduce the absorption of glucose (Shapiro and Gong 2002) whose effect can be observed in the increase of feed intake (Ordaz et al. 2017). Therefore, the objective of this research was to evaluate the effect of cactus (*Opuntia ficus-indica*) added to the lactating sows’ diet on voluntary feed intake and productive and reproductive performance post-weaning.

## Materials and methods

The study was conducted in the Pig farm of the Faculty of Veterinary Medicine and Husbandry of the Michoacan State Univesity of Saint Nicolas of Hidalgo. The farm is located at the municipality of Tarimbaro, Michoacán, Mexico at 19° 46′ 14.90″ NL and 101° 08′ 49.06″ WL and 1855 m above sea level. The municipality has a temperate climate, with an average temperature of 18.6 °C and average annual rainfall of 773.9 mm (INEGI 2010).

### Animals, diets, and housing

During a period of 12 months (from April 2016 to March 2017), data from 72 farrowings of 37 hybrid (Yorkshire × Landrace × Pietrain) sows (2.4 ± 1.1 farrowings sow^−1^ year^−1^) were collected; the sows’ age was expressed in parities and ranged between 1 and 4 farrowings. The sows were served at the time of estrus with hybrid boars (Yorkshire × Pietrain), and the pregnancy diagnosis was performed 21 days later; then, the pregnant sows were housed in corrals (16 m^2^) with capacity for six sows. During the first two thirds of gestation, the sows were fed daily with 2.0 kg of commercial feed (Table 1); in the last third of gestation (85–108 days of pregnancy), the sows received daily 2.5 kg of commercial feed; and the feed was served twice per day (8:00 and 14:00 h) into individual concrete feeders. The ingredients and the nutritional composition of the diet are shown in Table 1. The water supply was ad libitum using an automatic nipple drinker.

**Table 1:** Ingredient and nutrient composition of common gestation diet, common lactation diet (GC), and experimental lactation diet (GE)

Item	Gestation diet	Lactation diet	
		GC	GE
Ingredient (g/kg)			
Sorghum	824.0	649.7	649.7
Soybean paste	60.0	100.0	100.0
Canola paste	61.5	185.3	185.3
Orthophosphate	11.8	5.4	5.4
Calcium carbonate	14.0	12.4	12.4
Soy oil	22.0	38.5	38.5
Lysine	1.2	2.5	2.5
Salt	4.0	4.0	4.0
Vitamins and minerals premix^a^	2.0	2.5	2.5
Spineless cactus (*O ficus-indica*), %^b^			1.0
Nutrient composition^c^			
Metabolizable energy (Mcal/kg)^d^	3.3	3.3	3.3
Crude protein (%)	12.5	17.5	17.4
Crude fat (%)	3.7	4.5	4.4
Fiber (%)	3.1	4.3	4.5
Humidity (%)	12.0	12.0	12.8
Ash (%)	10.0	10.0	9.9
Calcium (%)^d^	0.75	0.75	0.75
Phosphorus (%)^d^	0.60	0.60	0.59
Lysine (%)^d^	0.52	0.95	0.94
Met-Cist (%)^d^	0.43	0.59	0.59

^a^Provided per kg of diet: Cu 30 mg; Fe 160 mg; Zn 160 mg; Mn 55 mg; Se 0.5; Cr 0.2 mg; vitamin A 14,200 IU; vitamin D_3_ 2800 IU; vitamin E 125 mg; vitamin K_3_ 5 mg; vitamin B_1_ 2.4 mg; vitamin B_2_ 8.7 mg; vitamin B_6_ 4.5 mg; vitamin B_12_ 0.05 mg; pantothenic acid 35 mg; folic acid 6 mg

^b^Spineless cactus supply was only in the morning fresh base. It was 1% based on body weight pre-farrowing sow throughout lactation phase

^c^To determine the nutritional composition of the diet with added of spineless cactus, the 1% of spineless cactus to the feed sample is added prior to bromatological analysis

^d^Calculated chemical concentrations using values for feed ingredients from the NRC (1998)

One week prior to the probable date of farrowing (day 109 of gestation), the sows were transferred to the farrowing and lactation area. In the farrowing and lactation area, two groups (G) were randomly arranged for the experimental design: control group (CG, *n* = 19 sows) in which 35 farrowings were analyzed and experimental group (EG, *n* = 18 sows) in which 37 farrowings were analyzed. Due to the capacity of the maternity area, there were six elevated cages for farrowing and lactation, and six sows were monthly monitored (three sows per group) from farrowing to weaning. Once that the sows from this area, CG sows were daily fed with 2.5 kg of commercial feed for lactating sows (Table 1). EG sows were daily fed with 1.0% cactus (on fresh basis according to sow’s live weight when entered to farrowing and lactation area) plus 2.5 kg of commercial feed for females in lactation each one (Table 1). After farrowing, commercial feed for lactating sows was offered ad libitum during 21 days of lactation in both groups; cactus supplementation continued in the case of EG.

The age of *O. ficus-indica* Cladodes, offered to EG sows, was approximately 90 days; the addition of 1% of *O. ficus-indica* was based on the established studies by Kritchevsky et al. (1988), Brahim et al. (2012), and Halmi et al. (2013) who by adding *O. ficus-indica* in a range of 1 to 5% report hypoglycemic effects in different species including humans; the decision in the taking of the lowest value was due to the volume of *O. ficus-indica* to the diet which could affect in less intake of commercial feed and due to the characteristics of the cladodes (deficiency in protein) could affect productive indicators of the sow. Then, the cladodes were stored at 4 °C until use. Before to supply the cladodes, they were chopped into approximately 3 × 2 cm pieces and immediately supplied to each sow from EG (at 8:00 h); at the same time, the commercial feed was offered to each sow. Every day this feeding activity was performed during the period that lasted the sow’s lactation and during the experimental phase.

**Fig. 1:**
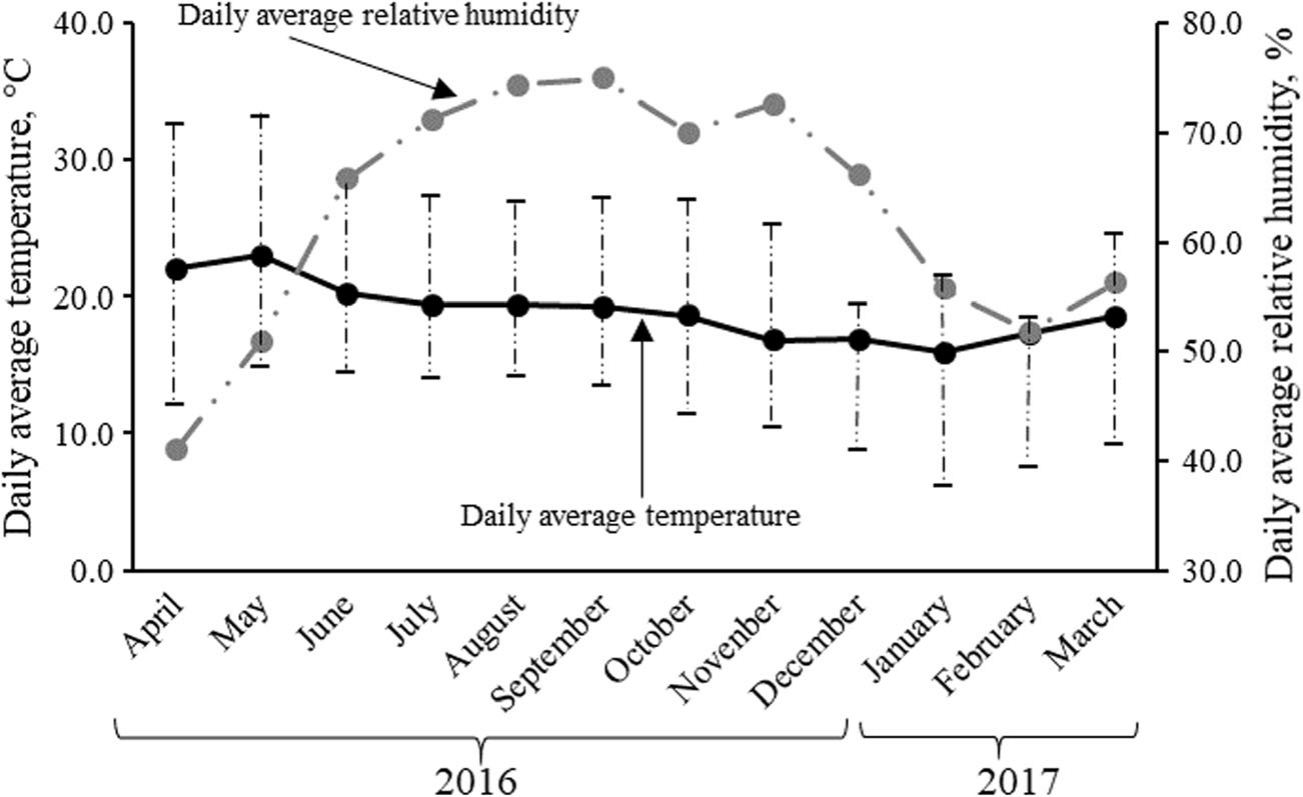
Environmental temperature and relative humidity recorded during experimental development

The sows from both groups were monitored and under the same management within the maternity area (farrowing and lactation stages). This area, as already mentioned, has elevated cages for farrowing and lactation (six cages); each cage has a stainless-steel bucket-type feeder (44.5 cm/width, 37.0 cm/height, and 33.0 cm/depth) and automatic nipple-type drinker. The temperature in the maternity area remained constant (18 °C) during the experimental period (temperature for sows and litters); for this, an infrared heater type Holme® brand with power from 750 to 1500 W regulated at 18 °C was used. The ventilation of the maternity was controlled through curtains. However, the use of curtains to control the ventilation could allow the climatic variations where it finds the production system (Fig. 1) to affect the temperature inside the maternity. In addition, in the area of service and gestation (semi-open confinement area), there is no control of the main climatic variables (temperature and humidity). For this reason, the year season effect on post-weaning reproductive variables was evaluated; since in this variable, many factors that modify the behavior of sows during and after lactation are immersed, such as feed intake, stress by hierarchical factors when confined to several sows of different ages and weights in a same lodging, and quality and quantity of diet offered (Ek-Mex et al. 2015), as well as physical-chemical changes in the composition of the *O. ficus-indica* cladodes (Pérez et al. 2015b).

### Experimental procedures

The variables evaluated were as follows: blood glucose (BG), daily (DFI), and total (TFI) feed intake during lactation, loss of body weight (LBW), weaning-estrus interval (WEI), and subsequent litter size (SLS). The determination of the BG was done by using a glucometer for human use (ACCU-CHEK Performan®) according to methodology described by Pérez et al. (2016). Blood sample takes were pre-prandial (8:00 h) and post-prandial (8:30 h), using puncture on right atrial vein on days 85, 100, and 110 of gestation; this is to avoid the appearance of confused effects associated with the presence of metabolic disturbances that present the sows during the gestation and that may affect the glycemia after initiating the monitoring of sows during lactation. In this regard, blood samples per sow in lactation phase were obtained on days 1, 3, 7, 10, 14, 17, and 21 of lactation. Days were determined according to Mosnier et al. (2010), who established that at day 85 of gestation, blood glucose concentration increases, and its decrease can be observed after the second week of lactation, while in the third week of lactation, the blood glucose levels become normal.

The sows of both groups were weighed (kg) at the time of entering the maternity area (7 days prior to projected farrowing) and until leaving it (21 days post-farrowing) with a fixed electronic scale (STG-1500-T1500SL, OCONY®/Mexico, with a capacity of 1–1500 kg). The individual DFI in each group was determined with a digital scale (Dibatec®, capacity of 40 kg and accuracy of ± 5 g); the food rejected per sow was daily weighed before the daily feeding of the sows. Data from DFI to compute TFI per sow in each group was used. At the end of lactation, LBW per sow in each group was computed using the following equations:

$$ {\displaystyle \begin{array}{c}{\mathrm{LBW}}_{\mathrm{kg}}=\mathrm{Sow}\ \mathrm{weight}\ \mathrm{pre}\hbox{-} \mathrm{farrowing}-\mathrm{Weaning}\ \mathrm{weight}\\ {}{\mathrm{LBW}}_{\%}=100-\left(\frac{\mathrm{Weaning}\ \mathrm{weight}\times 100}{\mathrm{Sow}\ \mathrm{weight}\ \mathrm{pre}\hbox{-} \mathrm{farrowing}}\right)\end{array}} $$

SLS per sow was obtained according to the group to which the sow belonged during the prior lactation (both CG and EG). However, all weaned sows, regardless of the group to which they belonged, were fed with the conventional diet (Table 1) during the subsequent gestation and managed in the same way.

### Statistical analysis

The data were analyzed by the methodology of fixed effects (MIXED) (SAS®). BG and DFI of the sows were analyzed using repeated measures (Littell et al. 1998) setting the sow as a random effect of time (days of lactation), and as fixed effects group, farrowing, day of lactation, season, the nesting farrowing within group and the interactions group × day of lactation and group × season. The model used was:

$$ {Y}_{ijklmn}=\mu +{G}_i+S{(G)}_{j(i)}+{FN}_k+{DL}_l+{S}_m+ FN{(G)}_{k(i)}+{\left(G\times DL\right)}_{il}+{\left(G\times S\right)}_{im}+{\left(G\times FN\times S\right)}_{ikm}\ {\upvarepsilon}_{ijklmn} $$

where *Y*_*ijklmn*_ = response variable: BG, DFI; *μ* = general average; *G*_*i*_ = fixed effect of the *i*th group with *i* = 1, 2; *S*(*G*)_*j*(*i*)_ = random effect of the *j*th sow nested with the *i*th group with *i* = 1, 2; *FN*_*k*_ = fixed effect of the *k*th farrowing number with *k* = 1, 2, 3, and 4; *DL*_*l*_ = fixed effect of the *l*th day of lactation with *l* = 1, 2, 3,…, 21, *S*_*m*_ = fixed effect of the *m*th season of lactation with *m* = spring, summer, autumn, and winter; *FN*(*G*)_*k*(*i*)_ = fixed effect of the nesting of the *k*th farrowing number within the *i*th group; (*G* × *DL*)_*Il*_ = fixed effect of the interaction of the *i*th group with the *l*th day of lactation; (*G* × *S*)_*im*_ = fixed effect of the interaction of the *i*th group with the *m*th season of lactation; (*G* × *FN* × *S*)_*ikm*_ = fixed effect of the interaction of the *i*th group with *k*th farrowing number and the *m*th season of lactation; and *Ɛ*_*ijklmn*_ = random error associated to each observation (~ NID = 0, *σ*^2^_*e*_).

The TFI, LBW, WEI, and SLS were estimated using group, farrowing number, season, the nestingof farrowing within group, and the interaction group × season as fixed effects and were taken as covariates sow weight pre-farrowing and litter size at weaning. The model used was the following:

$$ {Y}_{ijk l}=\mu +{G}_i+{FN}_j+{S}_k+ FN{(G)}_{j(i)}+{\left(G\times E\right)}_{ik}+{\beta}_1\left({X}_1-\right)+{\beta}_2\left({X}_2-\right)+{\upvarepsilon}_{ijk} $$

where *Y*_*ijkl*_ = response variable: TFI, LBW, WEI, and SLS; *μ* = general average; *G*_*i*_ = fixed effect of the *i*th group with *i* = 1, 2; *NP*_*j*_ = fixed effect of the *j*th farrowing number with *j* = 1, 2, 3, and 4; *S*_*k*_ = fixed effect of the *k*th season of lactation with *k* = spring, summer, autumn, and winter; *NP*(*G*)_*j*(*i*)_ = fixed effect of the nesting of the *j*th farrowing number within the *i*th group; (*G* × *S*)_*ik*_ = fixed effect of the interaction of the *i*th group with the *k*th season of lactation; *Β*_1_ (*X*_1_ *−* $\overline{X}$) = effect of the covariable weight of sow pre-farrowing; *Β*_2_ (*X*_2_ *−* $\overline{X}$) = effect of the covariable weight of litter at weaning; and *Ɛ*_*Ijkl*_ = random error associated with each observation (~ NID = 0, *σ*^2^_*e*_).

Differences between means used the method of leas squares means (LsMeans) with a *α* = 0.05. The values in the text are presented as least squares mean ± SD.

## Results

### Blood glucose levels in lactating sows

**Fig. 2:**
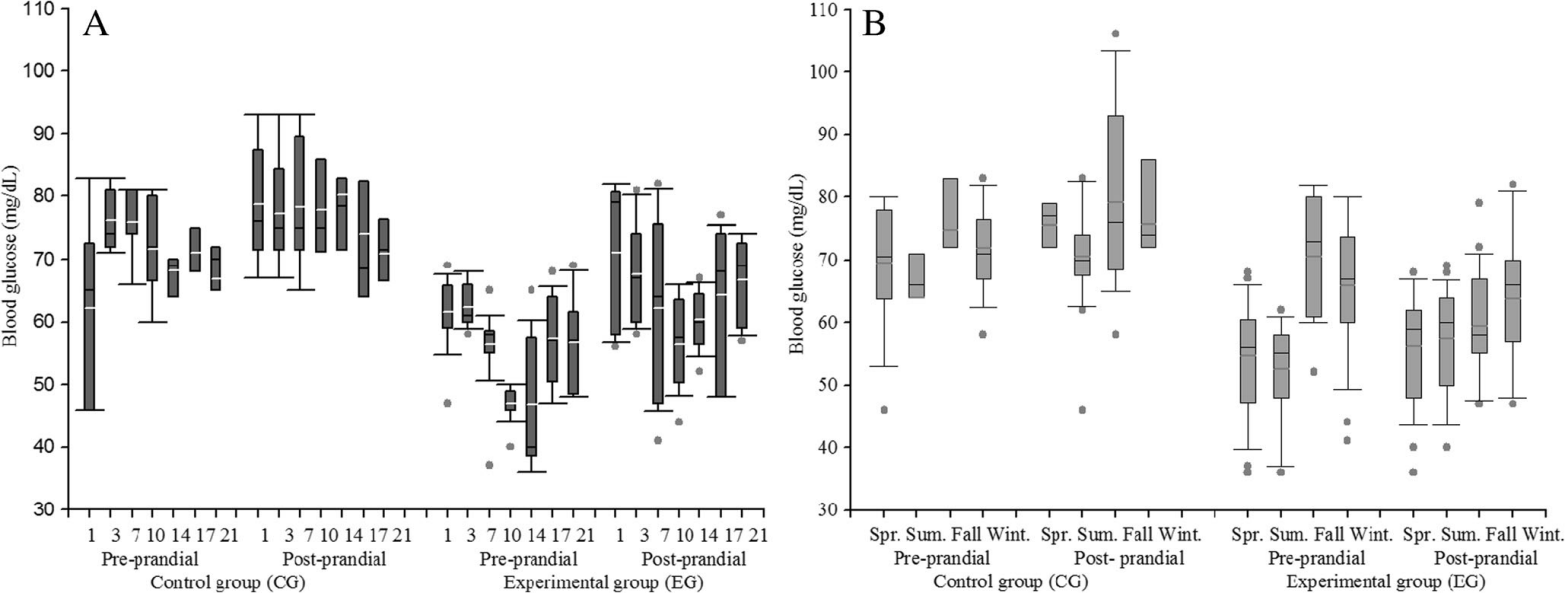
Least squares means for blood glucose levels of lactating sows according to interactions group × day (**a**) and group × season (**b**)

Effect of group on levels of BG pre- and post-prandial (*P <* 0.001) was found, as well as the interaction group × day of lactation (*P <* 0.001). Regarding the effect of group (*P <* 0.001), the levels of BG pre- and post-prandial EG were lower than CG (*P <* 0.05), BG pre-prandial 55.5 ± 8.5 vs 70.5 ± 8.4 mg dL^−1^, and BG post-prandial 65.3 ± 10.0 vs 77.3 ± 10.4 mg dL^−1^ for EG and CG, respectively. In relation to the effect of day of lactation (*P* = 0.010), the lower levels of BG were observed at the 10th day of lactation in EG, both for BG pre-prandial and post-prandial (Fig. 2).

According to the effect of the interaction between group × day of lactation (*P* < 0.001) on BG levels, blood levels observed of this metabolite were higher (*P <* 0.05) in CG in comparison to EG during the days analyzed (Fig. 2). Results that showed some consistency along the four seasons of the year analyzed (Fig. 2), while the levels BG pre- and post-prandial in fall and winter showed greater variability in EG compared to the rest of the seasons analyzed (Fig. 2).

The pattern of BG pre-prandial was affected by nesting FN (group) (*P* = 0.011) within CG; it was observed that sows of 1st and 4th farrowing showed the highest levels of BG, whereas when both groups were compared, sows of EG observed lower BG levels (*P* < 0.05) (Table 2). In relation to BG post-prandial, it did not observed a significant effect of FN (*P* = 0.792); however, a nested effect for FN (group) (*P* = 0.035) was observed on BG post-prandial levels. In this sense, the sows of EG showed lower levels of BG than CG sows (*P* < 0.05); CG observed BG post-prandial values that ranged from 75.7 to 80.3 mg dL^−1^, whereas EG observed BG post prandial values that range from 63.5 to 67.5 mg dL^−1^ (Table 2).

**Table 2:** Least squares means for blood glucose levels (mg dL^−1^) of lactating sows according to nesting farrowing number (group)

	Blood glucose pre-prandial		Blood glucose post-prandial	
Farrowing number	Control group	Experimental group	Control group	Experimental group
1	71.5b1 ± 9.3	53.8a2 ± 8.3	76.5a1 ± 11.6	63.5a2 ± 10.0
2	66.2a1 ± 7.4	54.6a2 ± 8.7	80.3a1 ± 7.4	66.0a2 ± 10.9
3	66.5a1 ± 6.9	56.0a2 ± 8.8	79.4a1 ± 9.9	65.0a2 ± 10.2
4	75.6b1 ± 5.2	58.1a2 ± 7.7	75.7a1 ± 8.0	67.5a2 ± 8.6

Different letters indicate statistical difference (*P* < 0.05) within column. Different numerals indicate statistical difference (*P* < 0.05) within row between blood glucose pre and post-prandial, respectively

### Feed intake of lactating sows

**Fig. 3:**
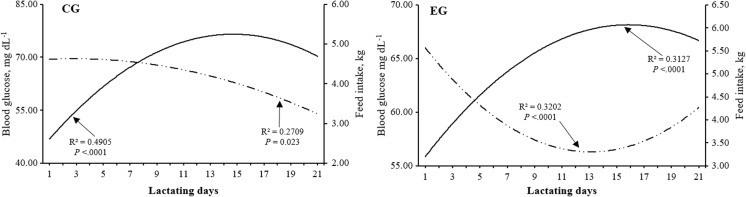
Blood glucose (BG) and daily feed intake (DFI) of lactating sows according to group

DFI was affected by the group, FN, season of the year (*P <* 0.001), and a nested effect FN (group) (*P* < 0.001), as well as by interactions of group × season of the year (*P* = 0.014) and group × week of lactation × FN. Within the interaction effect of group × week of lactation × FN on DFI, it was observed that in each of the evaluated weeks, EG observed a higher comercial feed intake (DFI) (*P* < 0.05), regardless of the consumption of cactus added to the diet when compared to the observed intake in CG sows (Table 3). According to the results of BG in EG, a relationship with the increase of DFI at the first week of lactation was observed, where such intake was 4.2 ± 1.5 vs 3.7 ± 1.4 kg commercial feed sow^−1^ in CG (Table 3). This feed intake (higher commercial feed intake per day) was similar in the rest of the weeks evaluated where EG showed the highest feed intake (Table 3). In this sense, it was observed that BG levels during the lactation affected DFI in both groups (Fig. 3). The results of linear regression for both DFI and BG levels were showed, whereas for each milligram per deciliter of BG increasing, the DFI increases in 0.025 g day^−1^ in the case of EG. In case of CG, it was observed that per each milligram per deciliter BG increasing, the DFI decreases in 0.020 g day^−1^ (Fig. 3).

**Table 3:** Least squares means for the average commercial feed intake per sow per week according to interactions group × week × farrowing number and group × week × season

	Week 1		Week 2		Week 3	
	CG	EG	CG	EG	CG	EG
Farrowing number						
1	3.0a1 ± 0.8	4.2b1 ± 1.1	3.8c1 ± 1.1	5.2d1 ± 0.7	4.5b13 ± 1.0	6.0e1 ± 2.1
2	4.5a2 ± 1.6	4.1b1 ± 0.9	4.9c2 ± 2.3	6.5d2 ± 1.6	5.3e2 ± 1.2	6.7d2 ± 1.2
3	3.4a3 ± 1.2	5.1b2 ± 1.0	4.9b2 ± 1.7	5.9c3 ± 1.1	4.9b3 ± 1.6	6.2d1 ± 1.1
4	3.1a3 ± 1.3	4.0b1 ± 1.2	4.0b1 ± 1.9	4.6c4 ± 1.2	4.2b1 ± 1.3	5.7d1 ± 2.1
Media general	3.7a ± 1.4	4.2b ± 1.7	4.3b ± 1.2	5.3c ± 1.5	5.0d ± 1.4	5.7c ± 1.3
Season						
Spring	3.4a1 ± 1.5	3.9b1 ± 2.0	4.2b1 ± 1.2	5.1c1 ± 1.6	4.6d1 ± 1.5	5.2c1 ± 1.9
Summer	4.2a2 ± 1.4	5.4b2 ± 1.7	5.2b2 ± 1.3	7.2c2 ± 1.3	5.5b2 ± 1.0	7.1c2 ± 1.1
Fall	4.0a2 ± 2.1	4.5b3 ± 1.1	4.2a1 ± 1.2	5.1c1 ± 1.4	4.6b1 ± 1.6	5.0c1 ± 1.7
Winter	4.1a2 ± 1.2	4.0a3 ± 1.0	4.0a1 ± 0.9	4.6b3 ± 1.7	4.2a3 ± 0.9	5.7c3 ± 1.1
Media general	3.9a ± 1.1	4.3b ± 1.5	4.6b ± 1.4	5.5c ± 1.4	5.1d ± 1.2	5.9c ± 1.2

Different letters on same line indicate statistical difference (*P* < 0.05) within row. Different numerals indicate statistical difference (*P* < 0.05) within column, for farrowing number and season of year, respectively

*CG* control group, *EG* experimental group

According to Fig. 3, it can be established that DFI by the sows of EG increased to 5.0 kg commercial feed sow^−1^ from the 2nd week of lactation, while the CG sows achieve that same intake after the second week of lactation. With respect to the effect of FN on DFI per week, higher DFI was recorded during the first week of lactation in sows of 2nd farrowing of the CG (*P* < 0.05): 4.5 ± 1.6 kg commercial feed sow^−1^, compared with the rest of the farrowings evaluated. In contrast, in EG, the weekly DFI was higher in each farrowinng evaluated with respect to CG; however, the third farrowing sows showed the highest intake of commercial feed (DFI) during each week of lactation evaluated (Table 3). Summer was the season of year where the minor (*P* < 0.05) DFI per week was registered (Table 3).

For the case of DFI average for 21 days of lactation of the CG sows, differences in the intake of commercial feed (*P* < 0.05) were observed in females of 1st farrowing (lower DFI), with respect to the sows of more farrowings (Table 4). Consequently, total commercial feed intake (TFI) per sow during the 21 days of lactation was higher in the EG in every analized farrowing (*P* < 0.05) (Table 4). However, the sows of the 4th farrowing, in both groups, showed the highest TFI (*P <* 0.05) (Table 4).

**Table 4:** Least squares means for average intake of feed and cactus (*O. ficus-indica*) per sow per day and total intake according to nesting farrowing number(group) and interacting group × season

	Feed intake and cactus average daily					Total intake of feed and cactus^a^		
	CG	EG			CG	EG		
FN	CF	CF	Cactus_FB_	Cactus_DB_	CF	CF	Cactus_FB_	Cactus_DB_
1	3.7a1 ± 1.3	5.1a2 ± 1.6	1.3a ± 0.5	0.15a ± 0.06	81.5a1 ± 11.5	102.7a2 ± 27.6	26.2a ± 7.2	2.0a ± 1.2
2	4.7b1 ± 1.4	5.1a2 ± 1.2	1.7b ± 0.5	0.20b ± 0.18	95.6b1 ± 9.5	102.9a2 ± 13.4	34.5b ± 9.1	3.8b ± 1.6
3	4.1c1 ± 1.2	5.3a2 ± 1.0	1.8bc ± 0.5	0.21bc ± 0.06	86.0c1 ± 12.7	107.3b2 ± 17.2	36.5c ± 6.5	4.1c ± 1.0
4	5.5d1 ± 1.2	5.4a2 ± 1.7	1.9c ± 0.6	0.24c ± 0.07	118.4d1 ± 7.1	123.1c2 ± 21.1	37.9c ± 8.9	4.2c ± 1.7
Media general	4.51 ± 1.3	5.22 ± 1.5	1.8 ± 0.7	0.20 ± 0.09	97.21 ± 8.3	108.62 ± 18	33.0 ± 6.7	4.2 ± 1.3
Season								
Spring	4.4a1 ± 1.2	5.2ab2 ± 1.5	1.6a ± 0.6	0.19a ± 0.18	89.7a1 ± 14.2	101.6a2 ± 22.7	32.8a ± 7.6	3.9a ± 1.4
Summer	4.4a1 ± 1.5	5.3ab2 ± 1.3	1.9a ± 0.4	0.24a ± 0.04	84.2b1 ± 14.8	100.5a2 ± 15.2	37.4c ± 5.7	4.4c ± 0.7
Fall	4.7a1 ± 1.8	5.7a2 ± 1.8	1.7a ± 0.6	0.22a ± 0.08	109.6c1 ± 10.5	121.8b2 ± 9.1	33.5b ± 7.0	3.6b ± 1.6
Winter	4.7a1 ± 1.5	5.0b2 ± 1.4	1.6a ± 0.6	0.19a ± 0.08	109.9c1 ± 10.3	117.2c2 ± 19.4	32.7a ± 6.7	3.6a ± 0.8
Media general	4.51 ± 1.9	5.32 ± 1.6	1.7 ± 0.6	0.20 ± 0.11	95.31 ± 9.5	110.62 ± 18	34.5 ± 8.7	4.5 ± 1.4

Different letters indicate statistical difference (*P* < 0.05) in same column for farrowing number and season of year, respectively. Different numerals indicate statistical difference (*P* < 0.05) in same row for commercial feed intake daily and total, respectively

*CG* control group, *EG* experimental group, *FN* farrowing number, *CF* commercial feed, *FB* fresh base, *DB* dry base

^a^Total intake during the 21 days of lactation

For aspects of the results of the consumption of cactus in fresh base, it was found that it oscillated between 26.4 and 37.9 kg sow^−1^ in 21 days of lactation. These amounts of cactus ingestion plus the intake of commercial feed (109.6 ± 18.0 kg) averaged a total intake of 160 kg sow^−1^ in 21 days of lactation, compared with 95.3 ± 9.5 kg total feed intake sow^−1^ in the CG. However, these average intakes were affected by the FN of the analyzed sows (*P* < 0.001). In this regard, first and second farrowing sows of both groups observed a lower TFI during the lactation (Table 4). In case of the effect of season of the year on TFI, CG sows in fall and winter showed a higher feed intake (109 kg in both seasons) (*P* = 0.014), whereas EG observed a higher commercial feed intake (121.8 ± 9.0 kg sow^−1^) in fall than the others seasons evaluated (*P* < 0.05) (Table 4).

### Loss of body weight of sows during lactation, weaning estrus-interval, and size of subsequent litter

In relation to loss of body weight (LBW) during lactation, the results of the nested effect of FN (group) determined that CG sows showed higher LBW than EG sows(*P* < 0.05); weight loss was higher in third farrowing sows CG (13.8 ± 2.2%) than the rest of the farrowings evaluated within this group and EG (*P* < 0.05). In the case of LBW in EG sows, this was higher in the fourth farrowing (6.9 ± 3.0%) in comparison to the rest of analyzed farrwings (*P* < 0.05). Nevertheless and independently of FN in both groups, an average 25.4 ± 10 kg LBW in CG at the end of lactation was observed, which represented 12.0 ± 4.0% of LBW. In the EG sows, the LBW average was 13.3 ± 12 kg or 5.9 ± 5.0% of weight loss at the end of lactation (Table 5). While LBW, according to interaction group × season, was higher in winter (*P* < 0.05) in CG sows, on average, these sows observed a LBW of 31.7 ± 13.0 kg or 12.0 ± 11.0% (Table 5). On the other hand, EG showed the biggest lost during lactation (12.4 ± 2.3 kg) in summer (*P* < 0.05) (Table 5), but when LBW observed as a percentage, it was 7.9 ± 5.2%.

**Table 5:** Least squares means for body weight pre- and post-farrowing and loss of body weight (kg and %) of sows according to nesting farrowing number (group) and interaction group × season

	Body weight pre-farrowing		Body weight post-weaning		LBW (kg)		LBW (%)	
FN	CG	EG	CG	EG	CG	EG	CG	EG
1	178a1 ± 14	180a1 ± 12	157a1 ± 13	168a2 ± 20	20.2a1 ± 8	11.2a2 ± 9	11.3a1 ± 4.7	6.5a2 ± 4.1
2	188b1 ± 15	209b2 ± 29	166b1 ± 15	197b2 ± 25	22.5a1 ± 7	11.1a2 ± 10	11.9a1 ± 3.6	5.1b2 ± 4.3
3	243c1 ± 32	217c2 ± 27	210c1 ± 30	205c1 ± 19c	33.5b1 ± 5	11.7a2 ± 8	13.8b1 ± 2.2	4.8c2 ± 2.5
4	237c1 ± 29	218c2 ± 31	211c1 ± 13	217d2 ± 27	28.1c1 ± 12	19.1b2 ± 10	11.8a1 ± 5.3	6.9a2 ± 3.0
Media General	2141 ± 27	2081 ± 26	1811 ± 22	1942 ± 21	23.41 ± 8	12.82 ± 9	11.71 ± 4	5.62 ± 4
Season								
Spring	203a1 ± 34	224a2 ± 39	183a1 ± 34	210a2 ± 33	20.4a1 ± 8.5	14.0a2 ± 17	10.1a1 ± 5.0	5.9a2 ± 6.7
Summer	229b1 ± 40	202b2 ± 20	201b1 ± 37	186b2 ± 20	28.2b1 ± 5.6	16.5a2 ± 12	12.4b1 ± 2.3	7.9a2 ± 5.2
Fall	194c1 ± 21	198b1 ± 26	174c1 ± 21	187b2 ± 21c	21.1a1 ± 3.4	10.8b2 ± 13	11.0a1 ± 1.9	4.5b2 ± 4.5
Winter	214d1 ± 29	218a1 ± 31	184a1 ± 27	207a2 ± 25	31.7c1 ± 3.1	12.0b2 ± 11	14.5c1 ± 5.2	5.2b2 ± 4.5
Media General	2101 ± 36	2111 ± 32	1851 ± 33	1982 ± 28	25.41 ± 10	13.32 ± 12	12.01 ± 4	5.92 ± 5

Different letters indicate statistical difference (*P* < 0.05) in same column for farrowing number and season, respectively. Different numerals indicate statistical difference (*P* < 0.05) in same row and into groups for body weight pre-farrowing, post-farrowing and LBW kilograms and percent, respectively

*CG* control group, *EG* experimental group, *FN* farrowing number, *LBW* loss body weight, *CG* control group, *EG* experimental group

**Fig. 4:**
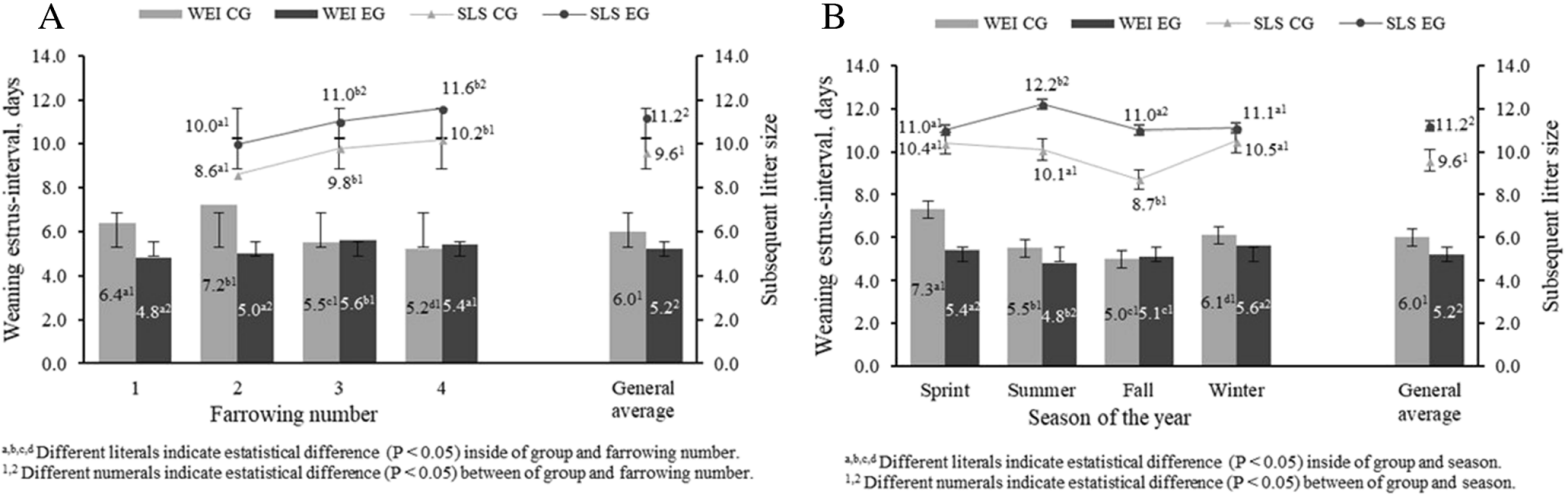
Least squares means for the weaning estrus-interval (WEI) and subsequent little size (SLS) according to nesting farrowing number (group) (**a**) and interaction group × season (**b**)

Finally, from the results of the reproductive variables analyzed in this research (weaning estrus-interval and subsequent litter size), effect of group (*P* < 0.001), nested effect FN (group) (*P* < 0.001), and interaction effect group × season of the year (*P* < 0.001) on these variables were found. In this sense, these effects determined that the EG sows obtained a less weaning estrus-interval (WEI), 5.2 ± 3.0 days, whereas the subsequent litter size (SLS) was 11.2 ± 1.1 piglet bigger than CG sows (*P* < 0.05) (Fig. 3). In addition, second farrowing CG sows observed major WEI (7.2 ± 1.5 days) than the rest of the evaluated sows (Fig. 4). It was also found that WEI was affected by season of the year (*P* < 0.001); such effect was greater in spring and winter in sows of both groups (*P* < 0.05). In spring, WEI was 7.3 ± 1.0 and 5.4 ± 1.2 days for CG and EG, respectively, whereas in winter, WEI was 6.1 ± 1.7 and 5.6 ± 1.1 days for CG and EG, respectively; these averages, within group, observed differences from each other (*P* < 0.05) (Fig. 4).

For SLS (Fig. 4), an increase in the number of piglets per litter in third and fourth farrowing sows in both groups was observed (*P* < 0.05). However, the EG sows showed a better performance in that variable (*P* < 0.05). There was also an interaction effect of group × season of the year on SLS (*P* < 0.001); in the summer, EG sows presented the largest SLS (12.2 piglets; *P* < 0.05) when compared to the rest of the seasons (Fig. 4) that showed similar averages among them (*P* > 0.05). In contrast, CG sows obtained a lower SLS average (9.6 piglets) than those obtained by EG (11.2 piglets); this variable was significantly affected by the seasons (Fig. 4); spring, summer, and winter registered a better SLS performance in CG sows (*P* < 0.05).

## Discussion

The decrease of BG in lactating sows fed with cactus as part of their diet (EG) (Fig. 2; Table 2) agrees with the effects reported (decrease in blood glucose) in human and other animal species after consuming the cactus (Halmi et al. 2013). It is suggested that this decrease in BG levels can be explained by the following assumptions: (1) mechanical, pectins, and mucilage present in the soluble fiber in cactus increase viscosity of feed, which makes the gut transit slower and increases the glucose absorption (Shapiro and Gong 2002) and (2) regulation of glucose metabolism; this assumption is associated to an increase of insulin concentration in plasma (28%) (Deldicque et al. 2013), which stimulates cells β-pancreatic provoking a reduction in blood glucose, through (i) oxidative decarboxylation, (ii) allosteric capacity to activate glutamate dehydrogenase, and (iii) transamination of *α*-ketoisocaprote (Halmi et al. 2013). Events subsequently increase the flow of the tricarboxilic acid and the reaction ATP/ADP, whose effect is reflected in the closing of potassium-ATP channels and depolymerization of the plasmatic membrane, allowing to open channels of Ca^2 +^ and then facilitate the secretion of insulin (Newsholme et al. 2005).

Although the decrease of BG pre-prandial was greater in the sows that intake cactus (EG) in comparison to CG sows (*P* < 0.05), the decrease of BG was greater at 10th day of lactation (*P* < 0.05), 47.0 ± 7.9 mg dL^−1^ in EG sows and 70.3 ± 8.7 mg dL^−1^ in CG sows (Figs. 2 and 3). These results show the possibility of manipulating the effect of lactational physiological hypophagia on lactating sows, specifically within the first week post-farrowing. Since, higher feed intake was observed in EG sows (4.2 ± 1.5 kg commercial feed sow^−1^ day^−1^) in the first week of the lactation (*P* < 0.05) in comparison to DFI of CG sows (3.7 ± 1.4 kg commercial feed sow^−1^ day^−1^) (Table 3). Likewise, the fact that FN did not affect the BG decrease in EG sows (*P* > 0.05) is an important finding. Mosnier et al. (2010) indicate that lactational physiological hypophagia has a greater effect on primiparous sows; they use nutrients from the feed not only for maintenance and milk production but also for their growth, because young sows have not still achieved their maximum body size (Pérez et al. 2015a). Therefore, when a reduced voluntary feed intake is due to the effect of lactational hypophagia, in this type of females, a greater effect is observed in LBW and post-weaning ovarian reactivation, as well as in subsequent fertility and prolificacy subsequent post-weaning (Mosnier et al. 2010).

Halmi et al. (2013) suggest that the non-fermentable dietary fiber of cactus increases the intestinal release of glucagon-like peptides-1 (GLP-1), which promotes an increase in insulin synthesis and inhibits the release of glucagon (Deldicque et al. 2013). In addition, the calcium content (2836.00 ± 157.71 mg 100^−1^ g, BS) of cactus (Villela et al. 2014) could stimulate the secretion of insulin by depolymerizing the plasma membrane of pancreatic cells with the increase of Ca^2+^ (Newsholme et al. 2005) and mitigate in this way physiological lactational hypophagia. In addition to the hypoglycemic effect, Pinos et al. (2010) pointed out that the fiber content (300 g kg^−1^) possibly produces a (i) mechanical capture of cholesterol and triglycerides, due to the formation of gel from the pectins contained in the cactus and (ii) exerted greater gastric distension in the sows. In this regard, our results indicate a greater volume of daily intake (*P <* 0.05) and therefore higher TFI at the end lactation (Table 4). Other studies had found that volumes of feed intake in lactating sows ranged from 92.8 to 103.3 kg in 21-day lactations (Cools et al. 2014). The results achieved with CG seem to agree with the study already mentioned (Table 4).

Several factors affect the sow’s TFI such as sow’s genotype, feed intake during pregnancy, body condition at farrowing, feeding frequency, water availability, age, metabolic physiology of the sow during lactation, as well as season of year (Soedea et al. 2001). Such aspects are in agreement with the present study (Table 4), due to the age of the sow (FN) and the season affected (*P <* 0.001) the TFI in both groups studied (Table 4). In this regard, the season effect on pre- and post-prandial BG levels (*P <* 0.001) increased BG level in fall and winter in sows of both groups (*P <* 0.05). Although EG observed lower levels of BG pre- and post-prandial compared to CG (Fig. 2), in those seasons, sows with higher body weight (third and fourth farrowing) were evaluated, which was reflected in a higher TFI during lactation (121.8 ± 9.1 and 115.2 ± 19.4 kg sow^−1^ of commercial feed in fall and winter, respectively), while total cactus intake was 33.5 ± 7.0 and 32.7 ± 6.7 kg sow^−1^ in FB in fall and winter, respectively (Table 4). In contrast, the lower BG levels of CG sows, in spring and summer, can be explained by the lower TFI of the sows (*P <* 0.05), at that time, because they had lower body weight (first and second farrowing) (Table 4).

Even when the temperature within the area of farrowing and lactation remained relatively constant (Fig. 1), in the area of service and gestation, it was not so (temperature was not controlled) and, therefore, the effect (*P* = 0.023) of the interaction group × season of year on the productive performance (feed intake, WEI, and SLS) was observed; in addition to that, there are other factors associated with the effect of season such as sanitary conditions, zootechnical practices, hierarchical interaction when weaned sows are confined in group, feed inputs, and quantity and quality of the diet, to mention some (Segura et al. 2015). In this sense, the results of this interaction showed that total intake of cactus in the summer by EG sows was higher (up to 12.5%) with respect to the other seasons evaluated (Table 4); it can be related to a higher body weight of pre-farrowing sow in summer (224 ± 20 kg) (*P <* 0.05) (Table 5). Since, the cactus added to the diet was according to the body weight of pre-farrowing sow. Likewise, the intake of cactus by the EG sows (33.0 ± 9.0 kg of cactus in FB) contradicts the hypothesis that the reduction of the feed intake of the lactating sows is due to the gastric capacity (Courboulay and Gaudré 2002). In addition to this, the intake of cactus (1.7 ± 0.6 kg average day^−1^ sow^−1^) was able to counteract the negative effects of lactational hypophagia by promoting greater intake of commercial feed in each farrowing and season evaluated (Table 4) and reduce the body weight loss (LBW) in EG sows during the lactation phase (Table 5).

Regarding to LBW related to FN, it was observed that regardless of FN analyzed, in EG sows the LBW was 5.8% compared with 12.2% of LBW recorded in CG (Table 5). However, third farrowing sows presented a LBW of 4.8%, (*P <* 0.05) compared to the rest of the farrowings evaluated in this group (Table 5). This lower LBW observed in third farrowing sows coincides with the highest TFI during lactation (107.3 ± 17.2 kg sow^−1^) of these sows (Table 4). With respect to the season, lower LBW was observed in EG sows in fall and winter (4.5 and 5.2%, respectively) (Table 5) which agrees with monitoring the sows with higher body weight (third and fourth farrowing), same with those who presented higher intake of commercial feed in such season and such group (121.8 and 115.2 kg during lactation, respectively). In addition, the cactus when reducing the BG (Table 2) favors higher feed intake (Ordaz et al. 2017) during lactation (Table 4) which reflects lower LBW (Table 5).

A greater intake of commercial feed and cactus of EG the sows could be improved, since the non-starchy polysaccharides contained in this cactaceous increase the viscosity of the food bolus (Chen et al. 2014). This higher viscocity reduces the speed of transit of the food bolus in the gastrointestinal tract (Mosenthin et al. 2001) and therefore generates greater nutrient absorption (Mosenthin et al. 2001). Regarding the absorption of nutrients, Chen et al. (2014) reported that the inclusion of pea fiber in the diet of pigs increased the expression level of the GLP-1 gene in the jejunum mucosa (*P <* 0.05). This gene has a key role in the renewal of intestinal epithelial cells and, in turn, modifies genes associated with digestive processes (Halmi et al. 2013). In this sense, cactus intake can also activate and increase the level of expression of the GLP-1 gene in the jejunal mucosa (Nuñez et al. 2013).

Returning to the effect of non-starch polysaccharides, when subjected to fermentation by the microbiota of the colon that leads to a greater production of volatile fatty acids (Cani et al. 2006), metabolites were intended for the body’s energy supply (Molist et al. 2009), which have a greater capacity for water retention at the intestinal level, essential for the catabolism of nutrients (Jha and Berrocoso 2010). Likewise, dietary fiber improves intestinal health; Jha and Berrocoso (2016) reported an increase in Lactobacilli (5.1%) and a lower number of coliforms (2.1%) with diets with beet pulp (50 g kg^−1^). Because of the fermentation of the pectin present in the pulp, this effect could be present in the sows that consumed cactus, due to the similarities in the type of fiber implemented in the investigations (Le Goff et al. 2002). Therefore, the non-starch polysaccharides, the greater time of the food in the gastrointestinal tract, as well as the greater renewal of the cells of the intestinal mucosa and the improvement of the intestinal health by effects of the dietary fiber present in the plant could promote lower LBW in EG sows (Table 5).

It has been determined that the LBW higher than 10%, during the lactation phase, negatively affects WEI, percentage of repeated services, and litter size in the next farrowing (Cools et al. 2014). Aspects were consistent with the results of this investigation, specifically those found in CG; in this group, the sows showed a higher overall WEI (6.0 ± 1.2 days, 144.0 h) (*P <* 0.05) compared to the EG sows (IDE = 5.3 ± 0.9 days, 127.2 h) (Fig. 4). In addition, EG sows showed in subsequent farrowing greater (*P <* 0.05) litter size (Fig. 4), compared with CG sows. It is possible that the effect of cactus on higher insulin synthesis (Newsholme et al. 2005) not only have been reflected in a higher feed intake and lower LBW (Table 4), but it was also reflected in the reproductive processes subsequent to the period of lactation. Since, insulin plays a key role in regulating reproductive processes of the sow (Soedea et al. 2001). An increased production of insulin is associated with higher synthesis of insulin-like growth factor-1 (IGF-1), which regulates the production of gonadotropic hormones such as follicle stimulating hormone (FSH) and luteinizing hormone (LH), essential for the production, maturation, recruitment, and ovulation of follicles (Ptak et al. 2006). Therefore, the effect that the cactus has on the glycolytic route could be acting as a modulator in the reproductive response of the sows in a favorable way by propitiating an earlier ovarian reactivation, which is reflected in a lower WEI and larger litter size in the subsequent farrowing, independently of FN and season of year (Fig. 4).

## Conclusion

The addition of cactus (*O. ficus-indica*) to the diet of lactating sows is a viable nutritional strategy to counter the effects of the lactational physiological hypophagia, because it can reduce the levels of blood glucose during the lactation, independently of the age of sow (farrowing number) or the season of year and. The reduction of this metabolite (blood glucose) causes an increase of voluntary feed intake of lactating sows with their respective consequences: lower loss body weight of these animals during the lactation phase, reduction of the weaning estrus-interval, and a greater size of litter in the subsequent farrowing.
